# Surgical Management of a Ruptured Giant Right Coronary Artery Aneurysm With Fistulization

**DOI:** 10.7759/cureus.69177

**Published:** 2024-09-11

**Authors:** Usman Aslam, Ujjawal Kumar, Ankur Gupta, Nikhil Iyengar, Zain Khalpey

**Affiliations:** 1 Department of General Surgery, HonorHealth, Phoenix, USA; 2 Department of Cardiothoracic Surgery, HonorHealth, Scottsdale, USA; 3 School of Clinical Medicine, University of Cambridge, Cambridge, GBR; 4 Department of Cardiology, HonorHealth, Scottsdale, USA

**Keywords:** adult cardiac surgery, cardiac tamponade, coronary artery ectasia (cae), coronary artery fistula aneurysm, giant coronary artery aneurysm

## Abstract

Coronary artery aneurysms (CAAs) are an uncommon condition with severe long-term consequences. We describe the surgical treatment of a right CAA that manifested as a compressive mass adjacent to the right atrium. A 60-year-old female patient presented with mid-sternal chest discomfort and a CT scan showing a 6.3cm x 5.5cm x 7cm mass along the anterior chest wall compressing the right atrium. Angiography revealed 95% proximal right coronary artery stenosis with contrast filling a giant CAA but no antegrade filling beyond the aneurysmal sac. While hospitalized, the patient experienced acute hypotension, and an urgent CT scan demonstrated interval bleeding into the pericardial sac with significant external compression of the right ventricular outflow. The patient was urgently taken to the operating room, where the right CAA was ligated at the neck and oversewn at the ostium. The patient developed a hemothorax on postoperative day 1 without a clear source of bleeding, but the remaining postoperative course was uneventful. Opportunities for surgery in patients with ruptured CAAs are rare due to the high pre-hospital mortality rate. Complex percutaneous coronary intervention is the preferred initial approach for asymptomatic CAAs, as was performed in this patient eight years prior. However, in the setting of acute tamponade, urgent operative intervention is the only viable management option. Aneurysmal rupture is an uncommon complication of CAAs that frequently leads to sudden death. This case demonstrates the successful management of an acutely ruptured CAA with urgent aneurysm ligation.

## Introduction

Coronary artery aneurysms (CAAs) are relatively uncommon, with a reported incidence of 0.3% to 5.3%, and are associated with poor long-term outcomes [1,2]. The first case of abnormal coronary artery dilation was described by Bourgon in 1812 [3]. Since then, numerous published studies have further stratified these focal dilations into two distinct groups: coronary artery ectasia (CAE) and CAAs. Dilation of a single coronary segment of at least 1.5 times the adjacent normal segment is defined as CAA, while diffuse lesions are termed CAE [4-6]. Approximately 20-30% of cases are congenital, while more than 80% of acquired cases are atherosclerotic in etiology. The right coronary artery (RCA) is the most commonly involved vessel [1,7]. CAAs are associated with a spectrum of clinical presentations, ranging from chest pain to sudden cardiac death due to obstructive coronary artery disease. The pathophysiology of acquired CAA is not well understood, but risk factors include coronary artery disease, certain vasculitis, and connective tissue disorders, as well as local coronary wall injury following intra-coronary manipulation [8-10]. Management strategies are tailored to the clinical manifestation, patient condition, and CAA characteristics, and may include medical therapy, percutaneous coronary intervention (PCI), or surgical intervention [11-13]. This report describes a rare case and the detailed surgical management of a right CAA rupture presenting as a compressive and fistulizing mass adjacent to the right atrium.

This case was presented as a poster presentation at the Eastern CardioThoracic Surgical Society meeting on October 8th, 2022.

## Case presentation

**Figure 1:**
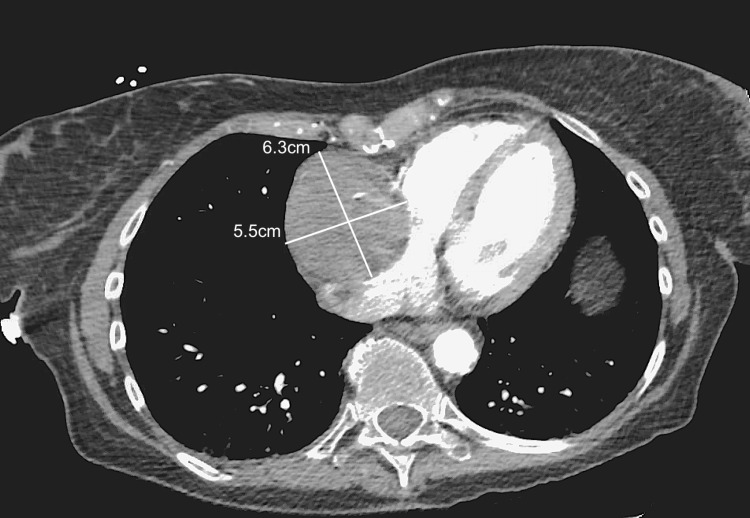
CT chest demonstrating a 6.3cm x 5.5cm x 7cm aneurysmal sac seen abutting the right heart wall with external compression on the right atrium.

**Figure 2:**
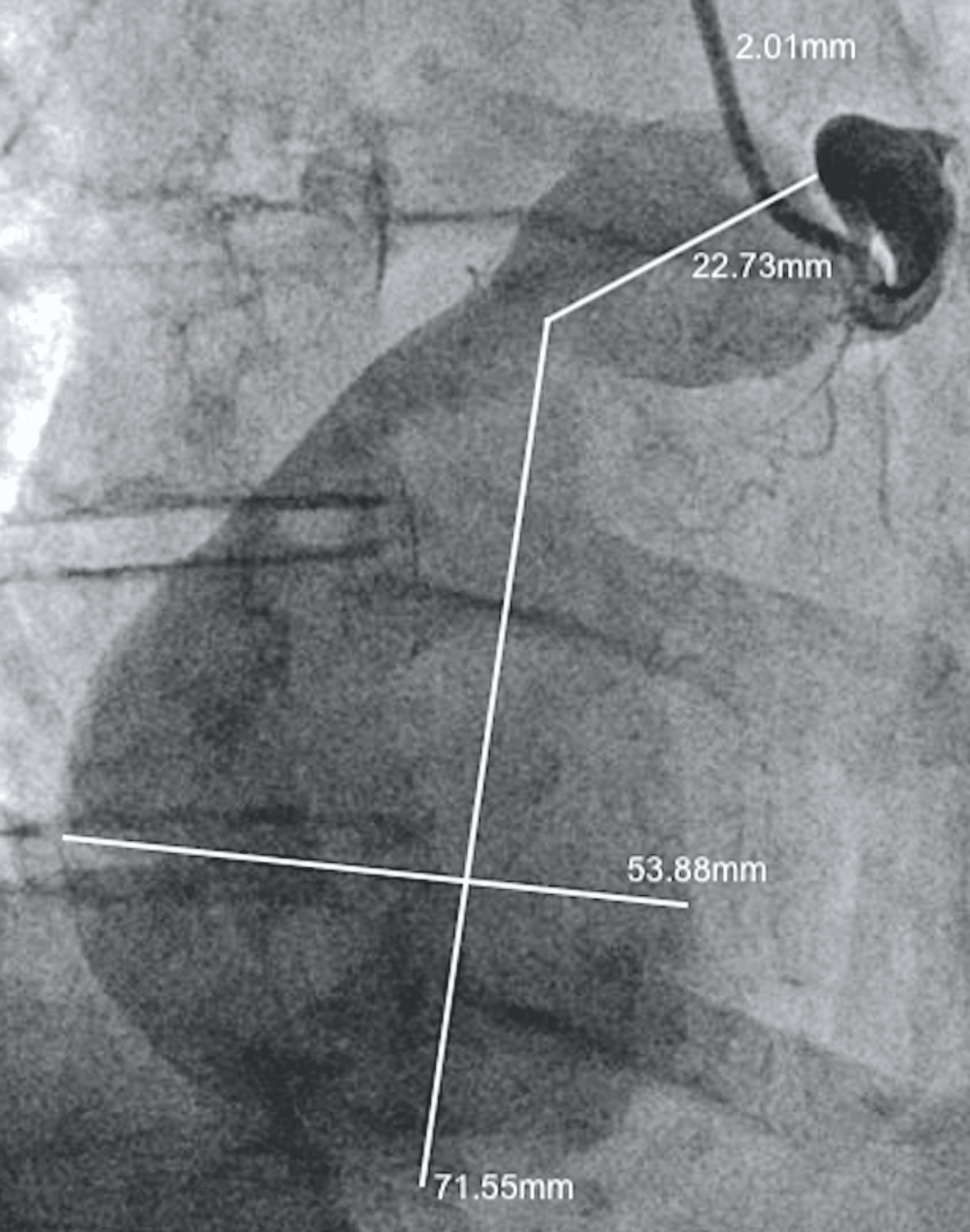
Giant right coronary artery aneurysm The angiographic evaluation showed an RCA with 95% stenosis followed by emptying into a massive aneurysm. Distal RCA system is seen filling retrogradely from left to right collaterals. No antegrade flow was observed beyond the aneurysmal sac. RCA: right coronary artery

**Figure 3:**
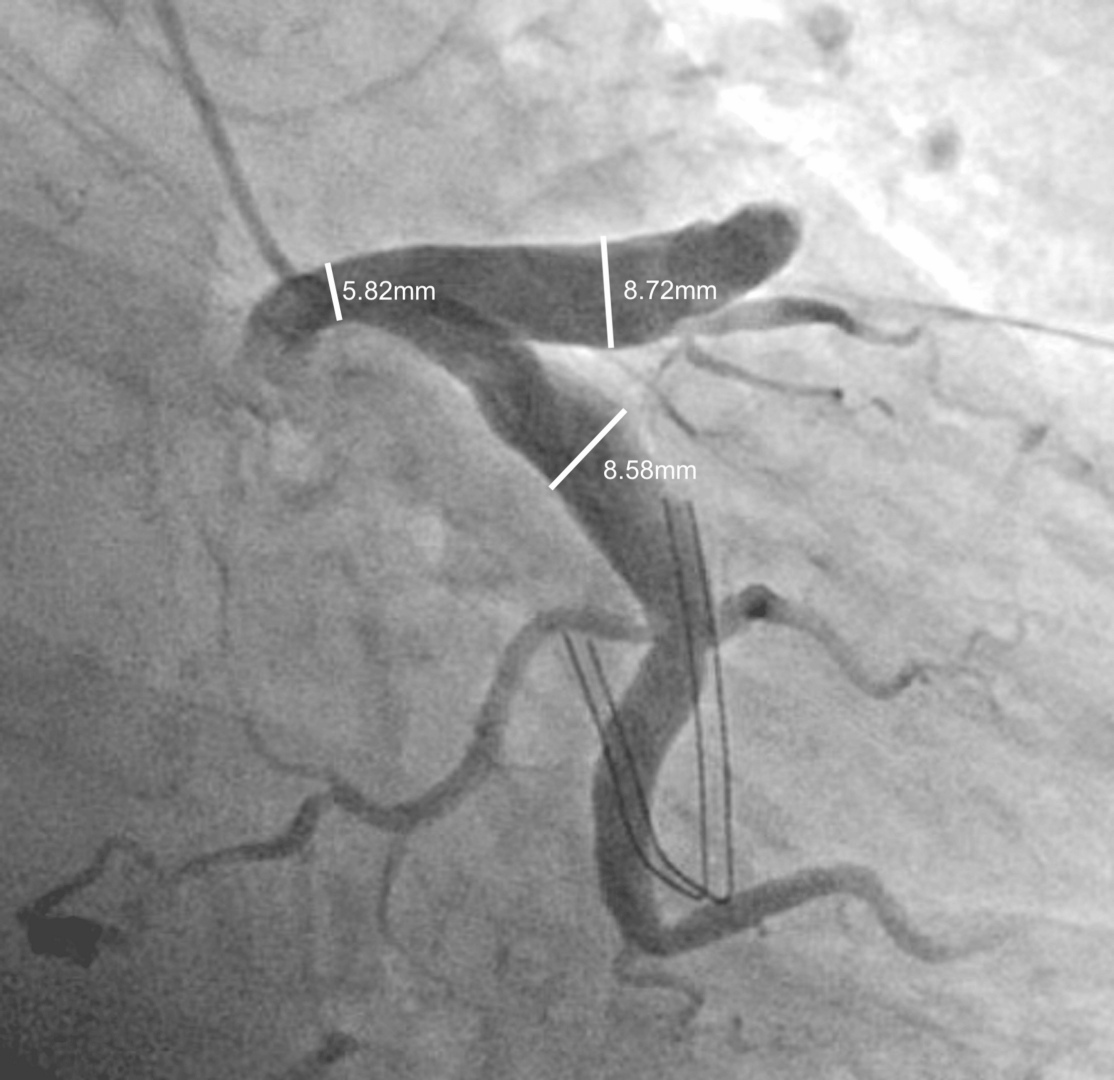
Severe ectasia of the left main, left circumflex, and left anterior descending arteries While there were no stenotic lesions seen in the left coronary circulation, slow flow was observed through the arterial system due to severe coronary artery ectasia.

A 60-year-old female patient with a history of chronic Coumadin use for Factor V Leiden, Lupus, Sjogren's syndrome, and coronary artery disease presented with acute-onset chest pain. The pain was described as a band-like pressure around the mid-chest, rated 7/10 in severity, and exacerbated by deep breathing or lying flat. Chest CT imaging revealed a 6.3cm x 5.5cm x 7cm mass along the anterior chest wall compressing the right atrium (Figure 1). The angiographic evaluation showed a 95% proximal RCA stenosis followed by emptying of contrast into a giant CAA with no antegrade flow beyond the aneurysmal sac (Figure 2). Retrograde filling of the distal RCA from the left heart was observed, with the left main, left anterior descending, and left circumflex arteries showing severe ectasia and slow flow throughout, but without any stenotic lesions (Figure 3).

**Figure 4:**
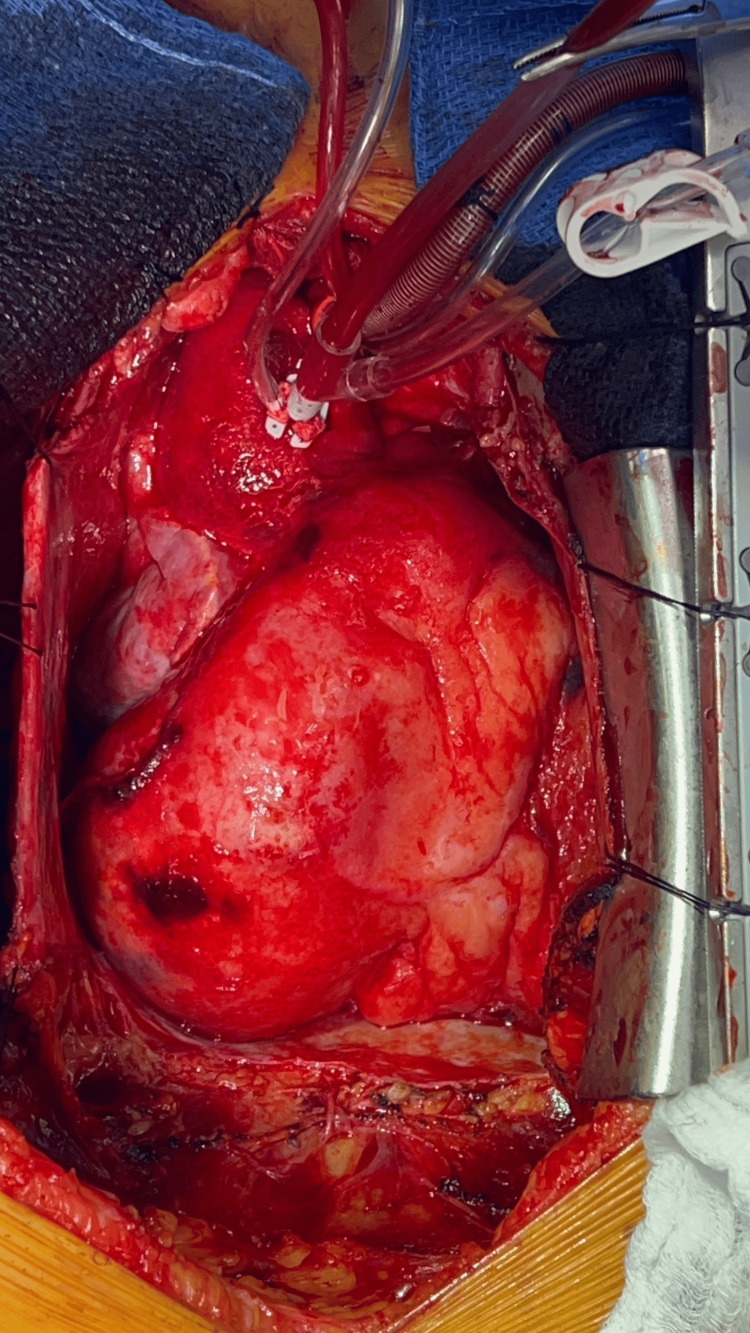
Giant coronary artery aneurysm originating from right coronary artery Intraoperative view of the heart showing the aortic bypass cannula in place. A giant coronary artery aneurysm is seen originating from the right coronary artery with a distal area of perforation.

**Figure 5:**
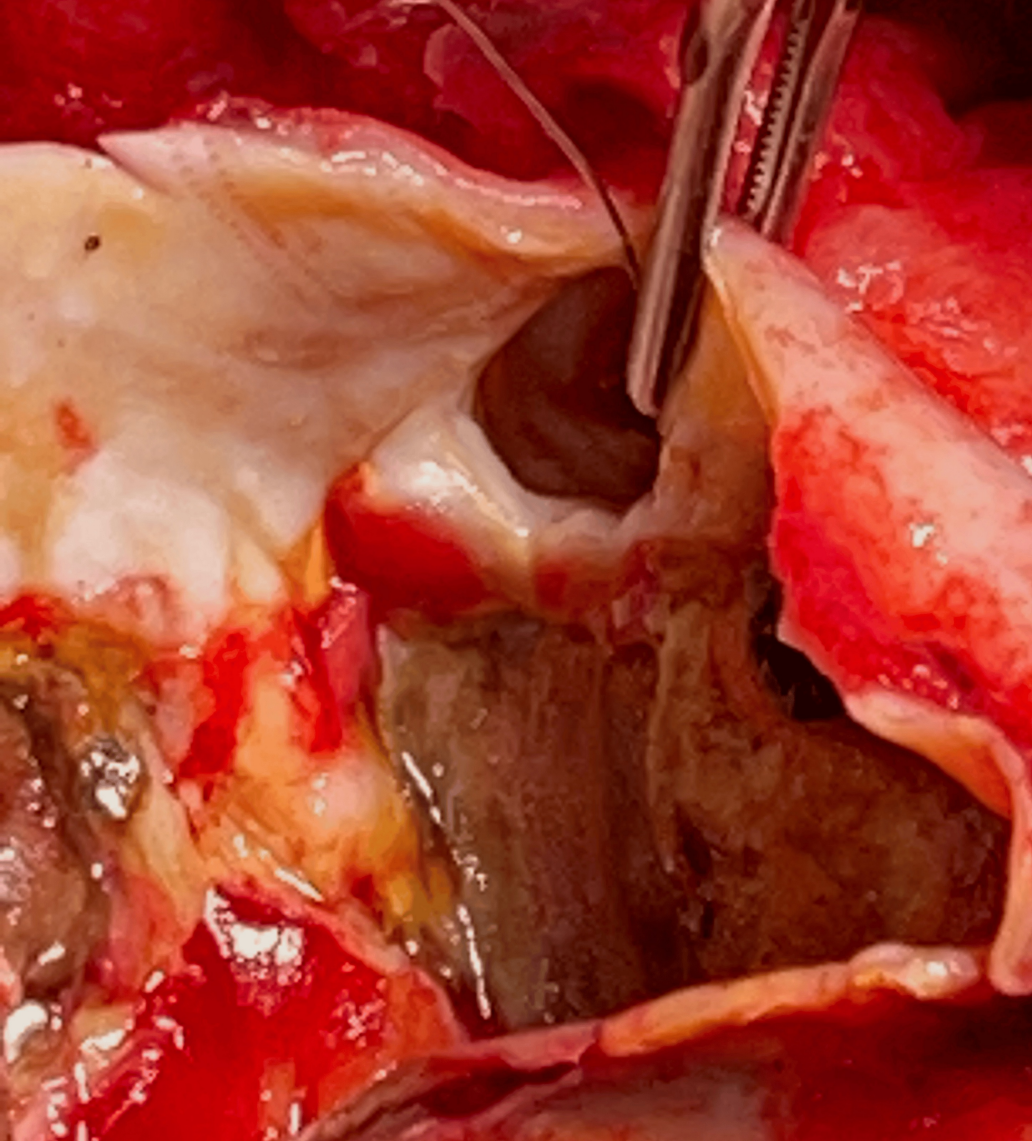
Right coronary artery ostium In this view, the aneurysm sac was opened and the right coronary artery ostium is seen entering the aneurysm sac.

**Figure 6:**
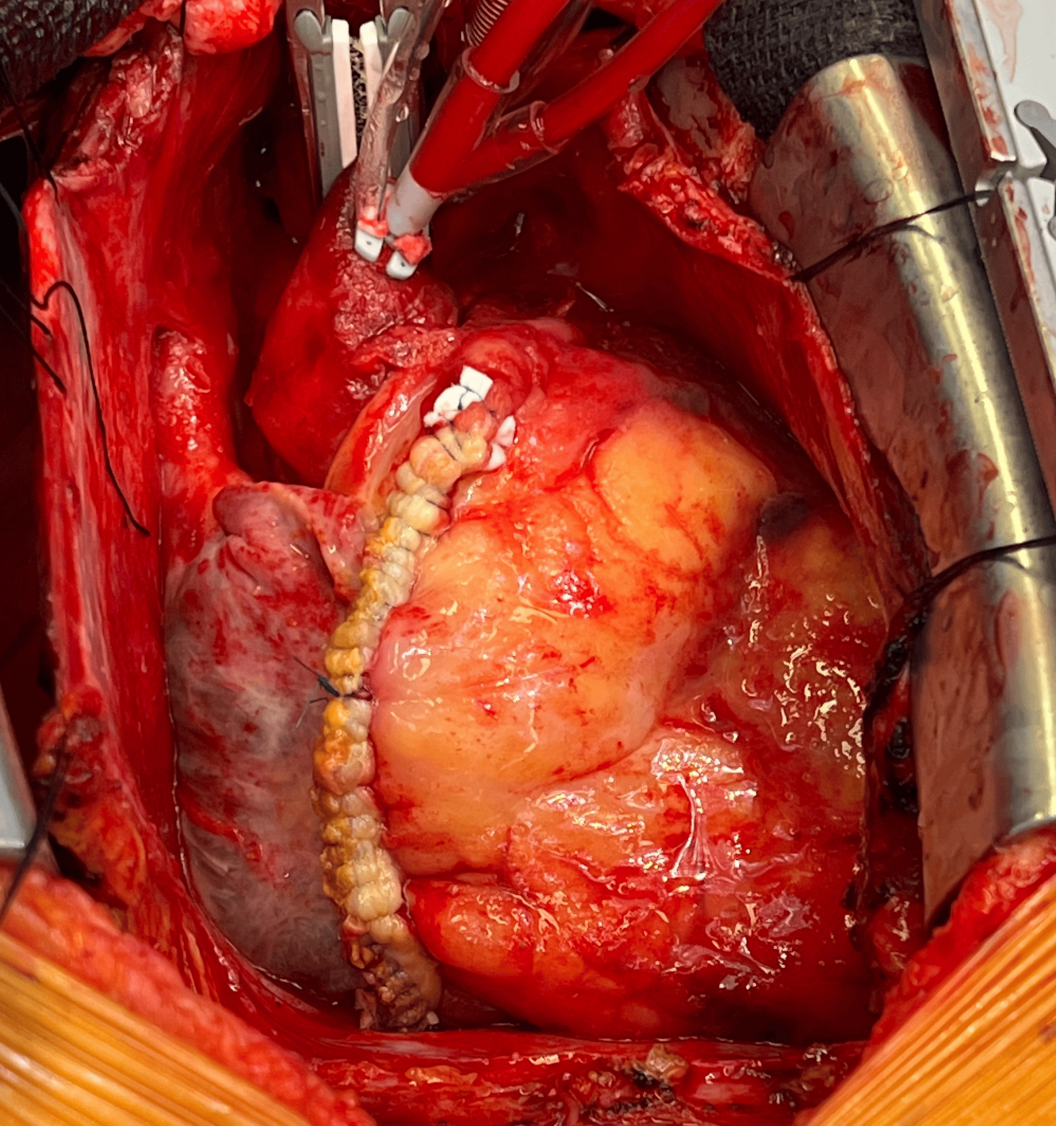
Complete ligation of the right coronary artery aneurysm sac was performed, extending from the neck of the ostium distally. The right coronary artery aneurysm was completely ligated and oversewn.

On the fifth day of hospitalization, the patient experienced hypotension, prompting urgent chest CT and transesophageal echocardiography. The aneurysm had enlarged to 5.8cm x 5.7cm, and there was an interval increase in pericardial blood. Significant external compression of the distal right ventricular (RV) outflow, pulmonic valve, and proximal main pulmonary trunk was observed. The patient was urgently taken to the operating room for repair of the ruptured CAA. A sternotomy was performed, and the patient was placed on cardiopulmonary bypass. Hemodynamic stability was maintained as 200cc of venous blood was drained from the pericardial sac. The RCA aneurysm (shown in Figure 4) was inspected, revealing that the RCA ostium was entering the aneurysmal sac (Figure 5). The aneurysm was ligated at the neck and ostium. A perforation in the anterolateral portion of the inferior aspect of the aneurysm, adjacent to an area of calcification, was identified as the likely source of the rupture. A fistula tract from the aneurysm to the right atrium was also ligated, with the complete surgical result shown in Figure 6. The patient was weaned off bypass without complication and transferred to the ICU on pressor support. Overnight, the patient was weaned off ventilator support without issue.

On postoperative day 1, a massive right hemothorax developed, and the patient was taken back to the operating room. The right hemithorax was washed out, and no bleeding was identified around the heart, suture lines, right atrium, or left atrium dome. The patient's remaining postoperative course was uneventful.

## Discussion

CAA represents an uncommon clinical condition characterized by a localized, abnormal dilation of the coronary artery, exceeding 1.5 times the diameter of the adjacent normal segments. In contrast, ectasia denotes a diffuse, dilated lesion involving 50% or more of the coronary artery length. The reported incidence of CAA varies, with most studies indicating a rate below 1% [1,2]. These clinical entities exhibit a wide spectrum of presentations, and management approaches are tailored to the specific clinical manifestations, patient factors, and aneurysm characteristics, as well as the experience and preferences of the treating physician.

The underlying mechanisms behind CAAs are not well understood. Weakened arterial walls resulting from chronic progressive inflammatory conditions, such as atherosclerosis in adults and Kawasaki disease in children, are believed to increase the risk of CAA development [8]. Stenotic coronary atherosclerosis and CAA frequently coexist, as they share several common histological features including hyalinization, lipid deposition, destruction of coronary layers, focal calcification, and fibrosis. In these scenarios, vasodilation induced by the release of nitric oxide further reduces coronary resistance to intraluminal pressure, predisposing the artery to dilation and CAA formation. While the coronavirus disease 2019 (COVID-19) pandemic has been observed to significantly increase the incidence of Kawasaki disease with cardiovascular complications in children and young adults, the relationship between COVID-19 and CAA remains unclear. Additionally, hereditary disorders like Marfan's syndrome and Ehlers-Danlos syndrome have been associated with CAA, and infectious processes that directly invade the vascular wall or provoke immune complex deposition may also contribute to its development [9].

Rupture of a CAA can lead to the formation of a contained hematoma and an intramyocardial mass. In patients receiving anticoagulation therapy, such a rupture may result in compression of the right heart and cardiac tamponade. These catastrophic cardiac events typically culminate in sudden death, myocardial infarction, or congestive heart failure. The bleeding into the pericardial sac not only exerts external compression on the heart, but the blood can also dissect along the planes of the ventricular spiral muscles, rupture into the cardiac chambers, cause wall motion abnormalities, and lead to ischemia by compressing adjacent coronary vessels.

The management of patients with CAAs remains controversial and there is a lack of high-quality evidence to guide the use of dual antiplatelet therapy or therapeutic anticoagulation. PCI focuses on restoring blood flow and the high thrombus burden seen in CAA often necessitates concurrent thrombectomy and glycoprotein 2b/3a inhibitor use. Despite these measures, no-reflow and distal embolization continue to be common complications [9]. Stent graft placement raises concerns, including the closure of adjacent side branches, stent thrombosis, and restenosis. Multi-center randomized trials have shown that expanded polytetrafluoroethylene (PTFE) stent grafts provide no improved outcomes compared to bare metal stents, and are associated with higher rates of restenosis and early thrombosis [12]. Patients with CAA undergoing coronary angiography have a higher five-year mortality rate than those without CAA [10]. Similarly, up to 54% of individuals with incidental CAA detected during pre-operative CT imaging experienced a major cardiovascular event (MACE) during long-term follow-up [13]. Given these challenges, a surgical approach may offer a safer and more reliable treatment option for many patients with CAA.

Surgical intervention is considered a viable alternative treatment strategy for managing symptomatic patients who are unsuitable for PCI, as well as those with large bifurcations or involvement of the left main coronary artery, complicated CAAs, and giant aneurysms at high risk of rupture [14]. The described surgical methods include aneurysmectomy with or without concomitant coronary artery bypass, aneurysm ligation, resection, and marsupialization [15]. However, the precise success rates of these surgical techniques remain unknown due to the rarity of these procedures and the potential impact of reporting bias. Ultimately, the optimal management of giant CAAs remains to be determined and requires a multidisciplinary approach involving cardiology, cardiac surgery, and diagnostic imaging specialists to evaluate the risks and benefits of various treatment strategies tailored to the individual patient.

## Conclusions

This case report describes a patient with a known right CAA that ruptured, a frequently fatal complication of CAAs. Eight years prior, the patient had experienced an acute RCA ST-elevation myocardial infarction. Coronary angiography revealed right-dominant perfusion and an ectatic RCA with complete occlusion of the proximal-mid vessel, including the RV marginal branch. Following complex PCI to the RCA without stent placement, the vessel was widely opened. Upon the patient's subsequent presentation, the aneurysm had worsened, with no antegrade flow beyond the distal end of the CAA, and a fistula had developed between the RCA and the right atrium. This fistulous segment was believed to be the site of rupture and subsequent cardiac tamponade. After both the aneurysm and the fistula were surgically ligated, adequate collateral flow from the left heart was observed. The optimal management strategy for giant CAAs remains elusive and necessitates a collaborative effort from a multidisciplinary team comprising cardiologists, cardiac surgeons, and diagnostic imaging experts. This team must carefully evaluate the risks and benefits of various individualized treatment approaches to determine the most suitable course of action for each patient.
